# General practitioner characteristics and delay in cancer diagnosis. a population-based cohort study

**DOI:** 10.1186/1471-2296-12-100

**Published:** 2011-09-26

**Authors:** Rikke P Hansen, Peter Vedsted, Ineta Sokolowski, Jens Søndergaard, Frede Olesen

**Affiliations:** 1 Research Unit and Section for General Medical Practice, Aarhus University, Bartholins Allé 2, DK-8000 Aarhus C, Denmark; 2 The Danish Cancer Society and the Novo Nordisk Foundation Research Centre for Cancer Diagnosis in Primary Care, Bartholins Allé 2, DK-8000 Aarhus C, Denmark; 3 Research Unit for General Practice, Aarhus University, Bartholins Allé 2, DK-8000 Aarhus C, Denmark; 4 Research Unit for General Practice, University of Southern Denmark, J.B. Winsløws Vej 9A, 1. DK-5000 Odense C, Denmark

**Keywords:** Cancer diagnosis, delay, GP characteristics, Denmark, family practice

## Abstract

**Background:**

Delay in cancer diagnosis may have serious prognostic consequences, and some patients experience delays lasting several months. However, we have no knowledge whether such delays are associated with general practitioner (GP) characteristics. The aim of the present study was to analyse whether GP and practice characteristics are associated with the length of delay in cancer diagnosis.

**Methods:**

The study was designed as a population-based cohort study. The setting was the County of Aarhus, Denmark (640,000 inhabitants). Participants include 334 GPs and their 1,525 consecutive, newly diagnosed cancer patients. During one year (September 2004 to August 2005), patients with incident cancer were enrolled from administrative registries. GPs completed questionnaires on the patients' diagnostic pathways and on GP and practice characteristics. Delay was categorised as patient-related (more than 60 days), doctor-related (more than 30 days) and system-related (more than 90 days). The associations between delay and characteristics were assessed in a logistic regression model using odds ratios (ORs).

**Results:**

No GP characteristics (seniority, practice organization, list size, participation in continuing medical education, job satisfaction and level of burnout) were associated with doctor delay. Patients of female GPs more often had a short *patient delay *than patients of male GPs (OR 0.44, 95% confidence interval (95%CI) 0.28 to 0.71). Patients whose GPs provided many services (OR 0.66, 95%CI 0.44 to 0.95) and patients attending GPs with little former knowledge of their patients (OR 0.68, 95%CI 0.47 to 0.99) more often experienced a short *system delay *than patients attending GPs with less activity and more knowledge of their patients. Patients listed with a female GP more often experienced a long system delay than patients of male GPs (OR 1.50, 95%CI 1.02 to 2.21). Finally, patients with low GP-reported compliance more often experienced a long system delay (OR 1.73, 95%CI 1.07 to 2.80) than patients with higher compliance.

**Conclusions:**

GP characteristics were not statistically significantly associated with doctor delay. However, some GP characteristics were associated with patient and system delay, which indicates that these factors may be important for understanding patient delay (e.g. perceived GP accessibility and the GP-patient relationship) and system delay (e.g. the GP's experience and opportunities for referring and coordinating diagnostic work-up).

## Background

Delay in cancer diagnosis and treatment is an important factor for prognosis, and it may also pose psychological problems for patients awaiting clarification of their disease [1-12]. In Denmark, general practitioners (GPs) act as gatekeepers to secondary care and they play a central role throughout the diagnostic interval [13-15]. Patients' care-seeking behaviour may be influenced by the GP-patient relationship [16]. Moreover, the GPs' personal characteristics and their professional skills may influence communication about symptoms and their diagnostic procedures, including the timing of referral for further investigation.

We have shown that 25% of newly diagnosed cancer patients experienced a total delay exceeding 168 days, though with large inter-individual variation [1,17]. Many studies have analysed how GP, practice, patient and structural characteristics affect clinical performance, including referral rates [18], imaging investigations [19] and other procedures, tests and follow-ups [20]. However, these studies have been able to explain only a small part of the observed variation in the length of delay.

It hence remains an open question whether some of the differences in the length of delay can be explained by factors relating to the GP and the practice setting. To our knowledge, this topic has not been addressed in previous studies.

The aim of this study was to analyse the associations between GP and practice characteristics and the length of different delay types in cancer diagnosis.

## Methods

### Study design

We conducted a cohort study in the County of Aarhus, Denmark. The county population totals 640,000 inhabitants with approximately 3,000 new cancer cases per year. Denmark's publicly funded health care system provides patients with free access to GPs and hospital care. More than 98% of Danish citizens are registered with a GP [21,22] who functions as a gatekeeper to the rest of the health care system, carrying out initial diagnostic investigations and referring patients to hospitals or outpatient clinics as needed. The average GP list encompasses approximately 1,600 patients.

The cohort included all newly diagnosed cancer patients in the County of Aarhus during the 1-year period from 1 September 2004 to 31 August 2005. (Hansen et al, submitted 2011). Patients were identified from the county's hospital discharge registry (HDR), which for each hospital admission and outpatient visit records the patient's unique civil registration number (CRN) [23], dates of admission and discharge and discharge diagnoses classified according to the International Classification of Diseases (ICD-10). We included all patients > 17 years with an incident cancer diagnosis documented in the HDR during the study period. We excluded patients with non-melanoma skin cancer and patients with a cancer recurrence. We linked the HDR data to the county's Health Service Registry (HSR) to identify each patient's GP.

### Data collection

Data were obtained from two questionnaires: a patient-specific questionnaire and a GP-specific questionnaire. Both were filled in by the GPs. We also obtained data from the HSR. In the patient-specific questionnaires, the GPs were asked to confirm the patient's diagnosis and to provide a detailed description of the patient's diagnostic pathway, complete with dates of reported symptoms, encounters, tests, referrals and involvement of other providers. The GPs filling in the questionnaires extracted relevant data from their own medical records and from hospital and specialist discharge letters. In addition, the GPs were asked about their knowledge about the patient and about patient compliance. In practices with more than one GP, we asked the GP most familiar with the patient to complete the questionnaire.

In the GP-specific questionnaire, the GPs answered questions about personal and practice characteristics, using, among others, the Warr-Cook-Wall job satisfaction scales [24], the Maslach Burnout Inventory [25] and items on working hours and participation in continuing medical education (CME) [26]. Non-responders received a reminder after three weeks. The GPs received a small economic compensation for their participation.

From the HSR we obtained practice-specific data about list size and volume of services provided (daytime surgery consultations, telephone consultations and home visits per 1,000 listed patients).

### Outcome measures

**Figure 1:**
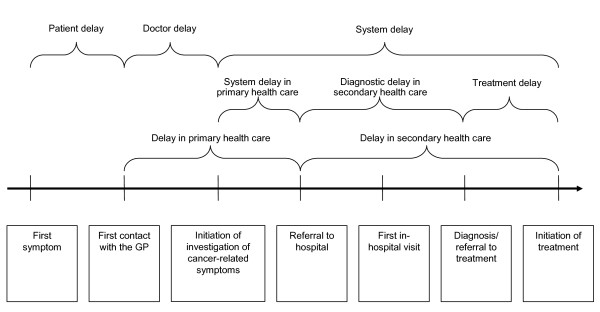
**Categorization of delay**.

Delay was computed from dates provided by the GPs and categorized by type as shown in Figure 1: *patient delay *(median 21 days, interquartile interval (IQI) 7 to 56), *doctor delay *(median 0, IQI 0 to 2) and *system delay *(median 55, IQI 32 to 93) [1] (Hansen et al, submitted 2011).

### Analyses

We only included GPs who had answered both questionnaires and who had been directly involved in their patients' diagnostic work-ups.

Emergency or out-of-hours cases and other activities falling outside normal GP working hours were excluded. Patient- and system-related delay were categorised as either short or long delay, with long delay defined as the 4^th ^quartile of all patients' delay (Hansen et al, submitted 2011). However, for doctor delay, we defined long delay as more than 30 days between the first encounter and the start of cancer-related investigations. We did so because the 75^th ^percentile for doctor delay was only 2 days. Such a definition of doctor delay (corresponding to the 91^st ^percentile) would allow the GP to have a time window of up to 30 days for watchful waiting to figure out the nature of new symptoms [27,28]. Thus, long patient delay was set to > 60 days, long doctor delay to > 30 days and long system delay to > 90 days.

We used multilevel random intercept logistic regression models with adaptive quadrature, using the Generalized Linear Latent and Mixed Model (GLLAMM) procedure to quantify whether GP and practice characteristics were associated with long delays. The hierarchical structure of GLLAMM allowed for non-independence of the explanatory variables, enabled clustering of patients within GPs and practices, and allowed for variability at patient, GP and practice levels [29-31]. Patients were nested within GPs, which were in turn nested within practices. The model assumes that the observations are conditionally independent at the lowest level given the higher level GP and practice random effects and the predictor variables. The analyses were adjusted for patient gender and age. We included all covariates in adjusted multivariate analyses after having tested for collinearity. The independent variables included were GP gender, years since graduation, practice organization, job satisfaction, burnout, CME history, working hours, list size, number of services per GP, the GP's knowledge of the patient and the GP's assessment of patient compliance; details are provided in Table 1. We measured the variance components at each level of the model (patients, GPs and practices), assuming that level 1 variance on the logit scale was π^2^/3, π^2 ^= 3.1416 [32]. The estimates are presented as odds ratios (ORs) with 95% confidence intervals (95%CIs). Additional analyses were performed after exclusion of gender-specific cancers (breast cancer and female/male genital cancers). Data were analyzed using Stata 11.

### Ethics approval

According to the Scientific Ethics Committee in the County of Aarhus, the project did not need approval by the Danish Biomedical Research Ethics Committee System. The study was approved by the Danish Data Protection Agency and the Danish National Board of Health.

## Results

In 2004, there were 458 active GPs in the County of Aarhus. Of these, 379 answered the questionnaire about GP characteristics (83%), and 410 GPs answered the patient-specific questionnaire (90%). Among these, a total of 334 (81%) completed the questionnaire about personal and practice characteristics. This group submitted 1,525 patient-specific questionnaires about diagnostic pathways for newly diagnosed cancer patients.

The associations between GP and practice characteristics and long delays at the patient level obtained from multilevel logistic regression GLLAMMs are described in Tables 1 and 2. GP seniority, practice organization, list size, CME activity, job satisfaction and burnout were not statistically significantly associated with any of the three delay types.

**Table 1:** Analyses of associations between general practice characteristics and delay in cancer diagnosis

GP characteristics		Patient delay; OR(95%CI)				Doctor delay; OR(95%CI)				System delay; OR(95%CI)			
		**N**	Unadjusted*	**N**	Adjusted*	**N**	Unadjusted*	**N**	Adjusted*	**N**	Unadjusted*	**N**	Adjusted*

Gender	Male	680(177)	1	571(147)	1	1021(100)	1	866(82)	1	766(198)	1	642(168)	1
	Female	314(51)	**0.54(0.38 to 0.77)**	230(32)	**0.44(0.28 to 0.71)**	490(39)	0.88(0.58 to 1.33)	368(32)	1.10(0.66 to 1.84)	374(106)	**1.38(1.00 to 1.89)**	278(87)	**1.50(1.02 to 2.21)**

Years since	≥ 20 years	735(169)	1	596(138)	1	1099(106)	1	901(88)	1	832(220)	1	671(183)	1
graduation	0-19 years	259(59)	0.96(0.69 to 1.35)	205(41)	1.06(0.68 to 1.64)	412(33)	0.83(0.54 to 1.27)	333(26)	0.81(0.48 to 1.37)	308(84)	1.11(0.79 to 1.55)	249(72)	1.11(0.75 to 1.63)

Practice	Solo practice	622(129)	1	485(99)	1	928(85)	1	732(64)	1	706(197)	1	553(159)	1
organization	Group practice	372(99)	**1.38(1.02 to 1.86)**	316(80)	1.18(0.82 to 1.70)	583(54)	1.00(0.68 to 1.45)	502(50)	1.13(0.71 to 1.80)	434(107)	0.82(0.59 to 1.13)	367(96)	0.93(0.64 to 1.34)

Job satisfaction	Satisfied	765(172)	1	619(138)	1	1155(101)	1	950(82)	1	877(239)	1	711(199)	1
	Not satisfied^1^	229(56)	1.12(0.79 to 1.58)	182(41)	0.93(0.61 to 1.41)	356(38)	1.21(0.80 to 1.83)	284(32)	1.32(0.81 to 2.15)	263(65)	0.86(0.60 to 1.23)	209(56)	1.00(0.67 to 1.49)

Burnout^2^	No	712(165)	1	577(133)	1	1116(102)	1	915(87)	1	845(233)	1	684(198)	1
	Yes	272(60)	0.95(0.68 to 1.33)	224(46)	0.79(0.53 to 1.18)	381(34)	0.96(0.63 to 1.47)	319(27)	0.91(0.55 to 1.51)	284(69)	0.86(0.60 to 1.22)	236(57)	0.83(0.56 to 1.22)

CME^3^	Yes	895(204)	1	722(159)	1	1368(128)	1	1124(108)	1	1023(277)	1	832(235)	1
	No	94(24)	1.20(0.73 to 1.96)	79(20)	1.19(0.67 to 2.10)	133(9)	0.70(0.34 to 1.44)	110(6)	0.56(0.22 to 1.40)	108(26)	0.87(0.51 to 1.48)	88(20)	0.95(0.52 to 1.73)

Working hours	≥ 40 hours/week	752(181)	1	645(149)	1	1141(110)	1	990(91)	1	854(234)	1	738(208)	1
	< 40 hours/week	191(36)	0.73(0.49 to 1.09)	156(30)	1.03(0.64 to 1.65)	293(25)	0.89(0.55 to 1.45)	244(23)	1.13(0.64 to 1.98)	221(52)	0.83(0.56 to 1.23)	182(47)	0.80(0.52 to 1.23)

List size^4^	< 1626(median)	508(108)	1	397(84)	1	743(72)	1	584(56)	1	558(156)	1	431(127)	1
	≥ 1626	475(114)	1.18(0.87 to 1.59)	404(95)	1.07(0.71 to 1.60)	751(66)	0.90(0.62 to 1.30)	650(58)	0.89(0.52 to 1.51)	566(143)	0.89(0.65 to 1.21)	489(128)	1.12(0.74 to 1.69)

Services^5^	< 9486(median)	520(117)	1	418(89)	1	748(72)	1	603(58)	1	563(168)	1	449(143)	1
	≥ 9486	463(105)	1.03(0.76 to 1.39)	383(90)	1.00(0.66 to 1.51)	746(66)	0.91(0.63 to 1.31)	631(56)	0.95(0.56 to 1.62)	561(131)	**0.72(0.53 to 0.98)**	471(112)	**0.66(0.44 to 0.99)**

Knowledge of the patient	Much	680(143)	1	609(129)	1	1045(91)	1	937(83)	1	773(220)	1	684(199)	1
	Little	312(85)	1.35(0.99 to 1.85)	192(50)	1.33(0.90 to 1.98)	463(48)	1.22(0.84 to 1.79)	297(31)	1.20(0.75 to 1.91)	365(84)	**0.72(0.53 to 0.99)**	236(56)	**0.68(0.47 to 0.99)**

Compliance	High	772(168)	1	713(151)	1	1186(105)	1	1100(95)	1	902(236)	1	830(219)	1
	Low	94(29)	1.60(0.99 to 2.58)	88(28)	1.63(0.99 to 2.68)	144(19)	1.51(0.88 to 2.60)	134(19)	1.63(0.93 to 2.85)	99(38)	1.58(1.00 to 2.51)	90(36)	**1.73(1.07 to 2. 80)**

Unadjusted and adjusted analyses of general practitioner and practice characteristics and the three stages of delay adjusted for patient clustering within GPs and practices. The N in each column is the number of answers with complete data. The number of patients with long delays is provided in brackets. Results are presented as odds ratios (ORs) with 95% confidence intervals (95%CIs).*ORs adjusted for patient gender and age.

^1^25% less satisfied

^2^Burnout index: emotional exhaustion score (0-38) > 26 and/or depersonalization score (0-24) > 9

^3^Participation in continuing medical education (CME). CME-group and/or supervision group

^4^Listed patients per GP according to the county's Health Service Registry (2003)

^5^Services (daytime surgery consultations, telephone consultations and home visits) per GP per year according to the county's Health Service Registry (2003)

**Table 2:** Analyses of associations between general practice characteristics and delay after exclusion of gender-specific cancers

GP characteritics		Patient delay; OR(95%CI)				Doctor delay; OR(95%CI)				System delay; OR(95%CI)			
		**N**	Unadjusted*	**N**	Adjusted*	**N**	Unadjusted*	**N**	Adjusted*	**N**	Unadjusted*	**N**	Adjusted*

Gender	Male	462(111)	1	389(93)	1	684(81)	1	582(65)	1	488(135)	1	416(115)	1
	Female	212(36)	**0.59(0.39 to 0.91)**	156(24)	**0.48(0.27 to 0.86)**	314(27)	0.72(0.45 to 1.16)	237(22)	0.91(0.49 to 1.69)	226(74)	1.32(0.91 to 1.91)	171(64)	**1.61(1.04 to 2.51)**

Years since	≥ 20 years	489(106)	1	395(87)	1	724(84)	1	593(69)	1	517(150)	1	421(125)	1
graduation	0-19 years	185(41)	0.99(0.65 to 1.50)	150(30)	1.05(0.61 to 1.79)	274(24)	0.74(0.46 to 1.21)	226(18)	0.72(0.39 to 1.35)	197(59)	1.07(0.73 to 1.57)	166(54)	1.16(0.75 to 1.78)

Practice	Solo practice	424(83)	1	328(63)	1	617(62)	1	482(44)	1	445(131)	1	349(107)	1
organization	Group practice	250(64)	1.40(0.96 to 2.03)	217(54)	1.31(0.83 to 2.07)	381(46)	1.21(0.80 to 1.84)	337(43)	1.49(0.87 to 2.54)	269(78)	0.97(0.68 to 1.39)	238(72)	1.10(0.74 to 1.63)

Job satisfaction	Satisfied Not	527(116)	1	425(92)	1	766(79)	1	631(62)	1	551(160)	1	454(137)	1
	satisfied^1^	147(31)	0.97(0.62 to 1.53)	120(25)	0.87(0.51 to 1.48)	232(29)	1.22(0.77 to 1.94)	188(25)	1.34(0.76 to 2.37)	163(49)	1.05(0.70 to 1.58)	133(42)	1.23(0.79 to 1.92)

Burnout^2^	No	481(109)	1	390(89)	1	732(80)	1	602(66)	1	526(158)	1	431(137)	1
	Yes	186(36)	0.83(0.54 to 1.28)	155(28)	0.68(0.41 to 1.12)	257(25)	0.87(0.54 to 1.40)	217(21)	0.88(0.49 to 1.59)	182(50)	0.89(0.60 to 1.33)	156(42)	0.88(0.57 to 1.35)

CME^3^	Yes	598(128)	1	483(101)	1	898(99)	1	741(81)	1	637(193)	1	527(167)	1
	No	72(19)	1.38(0.79 to 2.44)	62(16)	1.36(0.70 to 2.64)	84(7)	0.65(0.29 to 1.45)	78(6)	0.63(0.24 to 1.65)	72(16)	0.66(0.36 to 1.22)	60(12)	0.67(0.33 to 1.34)

Working hours	≥ 40 hours/week	517(117)	1	445(97)	1	774(86)	1	675(70)	1	552(166)	1	486(150)	1
	< 40 hours/week	122(24)	0.82(0.50 to 1.36)	100(20)	1.16(0.64 to 2.10)	179(19)	0.96(0.56 to 1.65)	144(17)	1.40(0.72 to 2.70)	128(33)	0.81(0.52 to 1.29)	101(29)	0.84(0.50 to 1.41)

List size^4^	< 1626(median)	347(75)	1	274(57)	1	496(61)	1	395(47)	1	354(111)	1	282(91)	1
	≥ 1626	317(67)	0.99(0.68 to 1.44)	271(60)	1.14(0.69 to 1.88)	491(46)	0.74(0.49 to 1.12)	424(40)	0.73(0.40 to 1.34)	351(95)	0.81(0.58 to 1.15)	305(88)	1.20(0.76 to 1.87)

Services^5^	< 9486(median)	345(78)	1	281(61)	1	494(58)	1	404(47)	1	349(121)	1	286(106)	1
	≥ 9486	319(64)	0.89(0.61 to 1.30)	264(56)	0.84(0.51 to 1.40)	493(49)	0.83(0.55 to 1.25)	415(40)	0.88(0.48 to 1.61)	356(85)	**0.59(0.42 to 0.83)**	301(73)	**0.53(0.34 to 0.83)**

Knowledge of the patient	Much	460(92)	1	409(82)	1	681(70)	1	613(62)	1	476(154)	1	426(141)	1
	Little	212(55)	1.37(0.93 to 2.01)	136(35)	1.55(0.95 to 2.53)	314(38)	1.20(0.78 to 1.84)	206(25)	1.31(0.77 to 2.23)	236(55)	**0.63(0.44 to 0.91)**	161(38)	**0.61(0.39 to 0.94)**

Compliance	High	519(109)	1	476(97)	1	769(82)	1	715(73)	1	561(162)	1	521(152)	1
	Low	74(20)	1.46(0.82 to 2.61)	69(20)	1.53(0.84 to 2.77)	111(14)	1.21(0.65 to 2.27)	104(14)	1.31(0.68 to 2.51)	73(28)	1.53(0.91 to 2.58)	66(27)	**1.80(1.04 to 3.13)**

Unadjusted and adjusted analyses of general practitioner and practice characteristics and the three stages of delay in the patient population ***after exclusion of gender-specific cancers ***adjusted for patient clustering within GPs and practices. The N in each column is the number of answers with complete data. The number of patients with long delays is provided in brackets. Results are presented as odds ratios (ORs) with 95% confidence intervals (95%CIs).*ORs adjusted for patient gender and age.

^1^25% less satisfied

^2^Burnout index: emotional exhaustion score (0-38) > 26 and/or depersonalization score (0-24) > 9

^3^Participation in continuing medical education (CME). CME-group and/or supervision group

^4^Listed patients per GP according to the county's Health Service Registry (2003)

^5^Services (daytime surgery consultations, telephone consultations and home visits) per GP per year according to the county's Health Service Registry (2003)

Patients attending a female GP more often experienced a short *patient delay *than those attending a male GP (OR 0.44, 95%CI 0.28 to 0.71) (Table 1).

Patients of GPs who provided many services more often experienced a short *system delay *than those attending GPs who provided fewer services (OR 0.66, 95%CI 0.44 to 0.99). A patient attending a GP with limited knowledge of this patient more often experienced a short *system delay *than a patient whose GP reported having good knowledge of the patient (OR 0.68, 95%CI 0.47 to 0.99). Cancer patients who attended a female GP more often experienced a long *system delay *than those attending a male GP (OR 1.50, 95%CI 1.02 to 2.21). Finally, patients with low GP-assessed compliance more often experienced a long *system delay *than patients with high GP-assessed compliance (OR 1.73, 95%CI 1.07 to 2.80).

As shown in Table 2 exclusion of patients with gender-specific cancers from the analyses did not change the results.

The amount of variation in delay at patient, GP, and practice levels obtained from the multilevel logistic regression models (GLLAMM) is presented in Table 3. There was no variation in patient delay at GP and practice levels. The variation in doctor delay and system delay at practice level was 7.11% and 5.71% respectively. After exclusion of gender-specific cancers there was some variation (8.4%) at practice level for doctor delay only.

**Table 3:** Source of variation in delay for persons residing at each level of models, obtained from Multilevel Logistic Regression Models (GLLAMM)

	Origin of variation		Variation^1^	Proportion of
Delay type	(level of model)	No. of units	(SE)^2^	total variation
**All cancer types**				
Patient delay	Patients (level 1)	801	π^2^/3	100,00
	GPs (level 2)	266	0.00 (0.00)	0,00
	Practices (level 3)	187	0.00 (0.00)	0,00

Doctor delay	Patients (level 1)	1234	π^2^/3	92,89
	GPs (level 2)	302	0.00 (0.00)	0,00
	Practices (level 3)	200	0.25 (0.20)	7,11

System delay	Patients (level 1)	920	π^2^/3	94,29
	GPs (level 2)	283	0.00 (0.00)	0,00
	Practices (level 3)	194	0.20 (0.13)	5,71

**After exclusion of gender-specific cancers**				
Patient delay	Patients (level 1)	545	π^2^/3	100,00
	GPs (level 2)	233	0.00 (0.00)	0,00
	Practices (level 3)	171	0.00 (0.00)	0,00

Doctor delay	Patients (level 1)	819	π^2^/3	91,60
	GPs (level 2)	276	0.00 (0.00)	0,00
	Practices (level 3)	194	0.30 (0.30)	8,40

System delay	Patients (level 1)	587	π^2^/3	100,00
	GPs (level 2)	250	0.00 (0.00)	0,00
	Practices (level 3)	182	0.00 (0.00)	0,00

^1^Level 1 variation was assumed to be π^2^/3 = 3.29, π^2 ^= 3.1416 (Snijders and Bosker, 1999)

^2^SE, standard error

## Discussion

### Main findings and implications

GP seniority, practice organization, list size, CME activity, job satisfaction and level of burnout were not associated with delay. It is remarkable that these factors, often hypothesised to be associated with practice performance and doctor delay, induced no delay.

It was also remarkable that patients attending female GPs more often experienced a short *patient delay *than patients attending male GPs. This finding raises the hypothesis whether female GPs are more accessible and trustworthy than male GPs [16]. *System-related delay *is composed of for instance waits for investigations in secondary care and delay due to administrative procedures or poor logistics in the planning of investigations. We found that having a female GP was more often associated with long *system delay *than having a male GP. The same was the case for patients with low GP-assessed compliance. Interestingly, the opposite was found for patients attending GPs providing many services or who had little knowledge of their patients as these patients more often had short *system delay*. These findings may be difficult to explain, but they invite the hypothesis that there may be differences in the quality of the GPs' function as care coordinators and case managers during an episode of diagnostic work-up [33].

We have not been able to identify studies on GP characteristics and delay in cancer diagnosis. We expected that a thorough examination of the included GP characteristics would establish an association with doctor delay. However, our findings suggest that differences in the GPs' diagnostic process, and thus differences in delay, should be examined with more specific measures addressing knowledge, attitudes and performance.

Our negative results raised the hypotheses that differences in GPs' delay may be explained by the perceived accessibility to GPs and about the quality of their care coordination. These hypotheses should be addressed in further research.

We were surprised to observe that patients of GPs with little knowledge of their patients more often experienced short system delays than those attending GPs with in-depth knowledge of their patients, but this is in line with a hypothesis raised earlier suggesting that continuity blinds GPs [34,35]. This should also be a topic for further exploration.

### Strengths and limitations of the study

We reduced selection bias by using complete registry data to identify potential patient study participants in the catchment area independently of participating GPs and hospital physicians. We were able to confirm patient eligibility by requesting that GPs validate the diagnoses. We may have underestimated the number of patients with long delays if non-participating GPs had relatively more patients with long delays. This may bias the study. However, as only a few GP and practice characteristics were clearly associated with delay, this potential source of bias may be of limited importance. Furthermore, the high response rate from the GPs (81%) reduces possible effects of selection bias.

We chose to dichotomize the variables in the study in order to try to find GP predictors for the different delay types and to analyse the data in a multivariate model. A presentation using continuous variables might have increased the power of the study, but would also have complicated the presentation of data. We used the dichotomization to find the most extreme cases of delay, which makes it possible to analyze whether extreme delays are more often seen for a specific group of GPs.

We found no association between *doctor delay *and several specific GP and practice characteristics. This may also be rooted in our definition of doctor delay which hides part of this information as we defined doctor delay as the 91^st ^centile (30 days) which might be too narrow; and the cut-off should perhaps be at a lower centile. On the other hand, one would expect that this extreme group should have shown us an association if there was any.

Using GLLAMM is advantageous in studies with clustered data (observations are not independent from each other) because it adjusts for the variation in long delay prevalence across patients, GPs, and practices. Moreover, it is important to measure the amount of variation in delay prevalence at different levels (i.e. patients, GPs, and practices) in order to identify potential intervention targets. None of the variation in delay was due to variation at GP level, and only a very small proportion was due to variation at practice level in some of the models.

As minimisation of recall bias is a prerequisite for valid findings, we encouraged the GPs to consult their electronic patient files when completing the patient-specific questionnaires. Misclassification is always a problem in studies where continuous variables are dichotomised with possible loss of information as a consequence. However, there is no standardised way to dichotomize e.g. delay and we believe that using the 75^th ^and 91^st ^centile, respectively, was the best way to test our hypotheses.

We assumed that patients reacted to serious symptoms as such rather than to symptoms assumed to relate to a specific, suspected cancer type. We further assumed that a doctor's sensitivity to initiation of appropriate investigation was a general physician trait rather than a trait associated with specific cancer types. For these reasons, we pooled the data for all cancer types, but we find it important to deepen our investigations in further studies exploring the same research questions in larger samples stratified for cancer diagnosis and for specific symptoms and groups of symptoms. The population-based approach and the homogeneous general practice structure throughout the country [22] make our results generalizable to other counties in Denmark or comparable health care systems, but differences in health care systems, especially levels of gatekeeping and cultural factors, should be considered before extrapolating our findings to other countries.

## Conclusions

There were no significant associations between factors such as GP seniority, practice organization, list size, CME activity, job satisfaction and level of burnout and doctor delay. We identified interesting GP gender differences in relation to patient delay that possibly reflect a better perceived accessibility to care when attending a female GP. A possible negative effect of knowing the doctor well should be further explored, and the same is the case for slight differences in system-related delay which may indicate doctor-dependent differences in the quality of care coordination.

## Competing interests

The authors declare that they have no competing interests.

## Authors' contributions

FO conceived the study. The study was conducted by RPH in consultation with all the co-authors, IS performed the statistic analyses in consultation with the other authors. RPH drafted the manuscript, and all authors contributed to revising the paper critically. Finally, all authors read and approved the submitted manuscript.

## Pre-publication history

The pre-publication history for this paper can be accessed here:

http://www.biomedcentral.com/1471-2296/12/100/prepub
